# Graphene Oxide–ZnO Nanocomposites for Removal of Aluminum and Copper Ions from Acid Mine Drainage Wastewater

**DOI:** 10.3390/ijerph17186911

**Published:** 2020-09-21

**Authors:** Carolina Rodríguez, Camila Tapia, Enzo Leiva-Aravena, Eduardo Leiva

**Affiliations:** 1 Departamento de Ingeniería Hidráulica y Ambiental, Pontificia Universidad Católica de Chile, Santiago 7820436, Chile; cnrodriguez@uc.cl (C.R.); erleiva@uc.cl (E.L.-A.); 2 Departamento de Química Inorgánica, Facultad de Química, Pontificia Universidad Católica de Chile, Santiago 7820436, Chile; camilatapiape@gmail.com; 3 Departamento de Química, Facultad de Ciencias, Universidad de Chile, Ñuñoa 7800003, Chile

**Keywords:** adsorption, heavy metals, removal, acid mine drainage, graphene oxide, zinc oxide, nanoparticles

## Abstract

Adsorption technologies are a focus of interest for the removal of pollutants in water treatment systems. These removal methods offer several design, operation and efficiency advantages over other wastewater remediation technologies. Particularly, graphene oxide (GO) has attracted great attention due to its high surface area and its effectiveness in removing heavy metals. In this work, we study the functionalization of GO with zinc oxide nanoparticles (ZnO) to improve the removal capacity of aluminum (Al) and copper (Cu) in acidic waters. Experiments were performed at different pH conditions (with and without pH adjustment). In both cases, decorated GO (GO/ZnO) nanocomposites showed an improvement in the removal capacity compared with non-functionalized GO, even when the pH of zero charge (pH_PZC_) was higher for GO/ZnO (5.57) than for GO (3.98). In adsorption experiments without pH adjustment, the maximum removal capacities for Al and Cu were 29.1 mg/g and 45.5 mg/g, respectively. The maximum removal percentages of the studied cations (Al and Cu) were higher than 88%. Further, under more acidic conditions (pH 4), the maximum sorption capacities using GO/ZnO as adsorbent were 19.9 mg/g and 33.5 mg/g for Al and Cu, respectively. Moreover, the removal percentages reach 95.6% for Al and 92.9% for Cu. This shows that decoration with ZnO nanoparticles is a good option for improving the sorption capacity of GO for Cu removal and to a lesser extent for Al, even when the pH was not favorable in terms of electrostatic affinity for cations. These findings contribute to a better understanding of the potential and effectiveness of GO functionalization with ZnO nanoparticles to treat acidic waters contaminated with heavy metals and its applicability for wastewater remediation.

## 1. Introduction

Water pollution with heavy metals is a worldwide concern. High levels of metals are toxic to both humans and the living organisms in aquatic ecosystems [1,2]. Metals come from either natural sources, like soil erosion and volcanic activities, or anthropogenic causes, as environmental problems derived from mining operations like acid mine drainage (AMD) [3,4]. For example, in Chile, where mining activities are concentrated in the northern regions, many rivers have high concentrations of heavy metals, which limits the potential uses of water [5,6]. Thus, new remediation alternatives are increasingly necessary to improve the quality and availability of water.

Conventional remediation technologies to remove metals from aqueous solutions are usually based on coagulation/flocculation, precipitation, oxidation, adsorption, ion exchange, and membrane filtration [7,8,9]. Among these methods, adsorption offers several advantages like flexible design and operation, high removal efficiency, cost-effectiveness and easy regeneration of the adsorbents [3,7]. Thus, several materials have been used for metals adsorption, such as zeolites [10,11], activated carbons [12,13], biochars [14,15], biomaterials [16], polymers [17], metal oxides [18,19], nanomaterials [20,21,22], and others. So far, nanomaterials have come to the forefront mainly due to their high specific surface area, which is known as one of the main factors determining adsorption magnitude [7,23].

Graphene oxide (GO) has been considered especially preferable as nano-adsorbent for heavy metals. Along with its large surface area, the abundant presence of oxygen-containing functional groups, such as ketones, epoxides, carboxyl, and hydroxyl groups, have been pointed out as the main reasons for its high efficiency adsorbing metal ions [24,25]. Thus, pristine GO has been shown to adsorb several metal cations such as sodium (Na), magnesium (Mg), cobalt (Co), cadmium (Cd), copper (Cu), iron (Fe), nickel (Ni), lead (Pb), strontium (Sr), and zinc (Zn) [26,27,28,29].

Several GO surface modifications have been studied to improve its adsorption capacity. The functionalization of GO by carboxylation (GO-COOH) increased the surface area and showed a better performance in Cu adsorption [28]. Moreover, magnetic graphene oxide (MGO) was proved for Co removal [30]. Graphene oxide functionalized with zinc oxide nanoparticles (GO/ZnO) has been recently studied and effectively accomplished to adsorb metal cations like Cu, Cd, Co, chromium (Cr), mercury (Hg) and Pb [31,32]. Zinc oxide (ZnO) nanomaterials have been shown to adsorb several metal cations such as Cu, Cd, manganese (Mn), Pb and Hg. However, ZnO nanoparticles usually are too small and unstable, needing to be impregnated into porous supports of larger size to form composite adsorbents [33,34]. For example, Alswata et al. [35] studied the use of zeolite as support for ZnO nanocomposites for the removal of Pb and arsenic (As) from contaminated waters, to improve the applicability of ZnO and avoid the agglomeration of nanoparticles. Although GO and GO/ZnO nanocomposites have been reported as efficient metal adsorbents, there is still little research on their ability for metal cation adsorption under acidic conditions.

Aluminum (Al) and copper (Cu) are two typical AMD pollutant ions, which could be found at high concentrations naturally or due to human activities [36,37,38]. Copper is an essential micronutrient and generally occurs as a highly mobile divalent cation under acidic conditions [39,40,41]. At high concentrations, Cu can alter human health, causing anemia, liver and kidney damage, and stomach and intestinal irritation [42]. On the other hand, Al is highly soluble at low pH and generates toxicity in the aquatic environment [43]. The rivers in northern Chile have registered a large increase in the concentration of heavy metals due to the effects of mining activity [44]. Indeed, some studies have reported high concentrations of Cu, Zn, Fe, Al, among other metal ions, which restricts the potential uses for these waters [45,46]. Particularly, high concentrations of Al and Cu have been observed in the rivers such as Toro, Malo, Vacas Heladas and Yerba Loca creek in north-central Chile [39,46,47]. In this way, emerging and efficient technologies to remove these pollutants (Al and Cu) would bring great benefits to increase the availability of quality water resources, especially considering future scenarios of water scarcity.

Although GO has been extensively studied, there are few studies on the functionalization of GO with ZnO for heavy metal removal under acidic conditions. In fact, only two include the study of Cu, but none have investigated the removal of Al or some other trivalent ion. Here, we present the preliminary results about the adsorption ability of GO and GO/ZnO nanocomposites for Al and Cu removal from synthetic AMD waters. The adsorbents were characterized previously to batch adsorption tests. Experimental data were fitted using Langmuir and Freundlich model isotherms in adsorption experiments with different pH conditions. The goal of this research was to explore the potential of GO/ZnO, a little-studied functionalization for the adsorption of metal ions, as a remediation alternative for the removal of Al and Cu from AMD waters, which have commonly acidic pH values.

## 2. Materials and Methods

### 2.1. Materials

All the reagents and solvents used were of analytical reagent grade. Graphite powder was purchased from Merck. ZnO nanoparticles, NaNO_3_, KMnO_4_, CuSO_4_·5H_2_O, KAl(SO_4_)_2_·12H_2_O, and MnSO_4_·H_2_O were purchased from SigmaAldrich. Sulfuric acid (H_2_SO_4_), hydrochloric acid (HCl), hydrogen peroxide (H_2_O_2_), and absolute ethanol were purchased from Merck. All solutions were prepared with deionized (DI) water.

### 2.2. Preparation of Nanoadsorbents (GO and GO/ZnO)

GO was prepared using a modified Hummers method [48], which uses additional KMnO_4_. Briefly, concentrated H_2_SO_4_ (300 mL) was added to a mixture of graphite powder (9 g) and NaNO_3_ (4.5 g), and the mixture was cooled using an ice bath to 0 °C. KMnO_4_ (27 g) was added slowly in portions to keep the reaction temperature below 20 °C. The reaction was warmed to 35 °C and stirred for 7 h. Additional KMnO_4_ (27 g) was added in one portion, and the reaction was stirred for 12 h at 35 °C. The reaction mixture was cooled to room temperature and poured onto DI-water ice (400 mL) with 30% H_2_O_2_ (3 mL).

The mixture was then washed in succession with a 10% HCl solution; at each wash, the mixture was filtered using a 0.45 µm membrane filter. The filtrate was centrifuged (6000 rpm for 20 min), and the supernatant was decanted away. The solid resulting from the filtration and centrifugation processes was dialyzed against DI water for 7 days to remove residual salts and acids. After that, the suspension was diluted 1:2 and stirred (Magnetic Stirrer SH-2, Huanghua Faithful Instrument Co., Ltd., Huanghua City, China) overnight. The resulting solution was bath sonicated (Isolab Laborgerate GmbH, Wertheim, Germany) for 30 min at 40 kHz and centrifuged for 40 min at 6000 rpm (Hermle Z 206 A, Wehingen, Germany) to obtain single-layered GO. Finally, the suspension was oven-dried at 70 °C for 4 h.

Commercial ZnO nanoparticles (average size of 100 nm) were used for the preparation of GO/ZnO nanocomposites. ZnO nanoparticles were dispersed into absolute ethanol (0.33 mg/mL), and then the solution was sonicated for 40 min. After sonication, 300 mL of ZnO solution was carefully added drop by drop on 200 mL of GO dispersion (2.5 mg/mL). The mixture was placed in a water bath to eliminate the ethanol. Finally, the solid GO/ZnO nanocomposite was obtained after oven-drying at 70 °C for 4 h.

### 2.3. Nanoparticles Characterization

The three nanoparticles, GO, ZnO and GO/ZnO, were subjected to characterization prior to adsorption experiments. Fourier transform infrared (FT-IR) spectra (KBr pellet method, 400–4000 1/cm) were obtained on a FT-IR 4600 Spectrometer (JASCO, USA) at a resolution of 1 1/cm. The specific surface areas of the samples were determined by Brunauer–Emmet–Teller analysis (BET) N_2_ adsorption–desorption analysis (Micromeritics Instruments Corp., Norcross, USA). The surface morphology was determined by using a scanning electron microscope (SEM) (JSM-IT300LV, JEOL, Japan) coupled with energy-dispersive X-ray spectroscopy (Oxford Instruments, SEM-EDX, High Wycombe, UK) which was carried out on a representative area of a homogenized sample. To measure the pH of point of zero charge (pH_PZC_), we prepared 40 mL solutions at different pH values (3 to 11) adjusted with NaOH 0.1 M and HCl 0.1 M [49]. Then, 30 mg of nanoparticles samples were added to these solutions and shaken at 300 rpm at room temperature for 48 h. The final pH was measured and plotted against initial pH, and the intersection of this curve with the line that represents pH initial = pH final determines the pH_PZC_ value.

### 2.4. Batch Adsorption Experiments

Batch experiments were conducted to obtain the equilibrium isotherms separately for Al(III) and Cu(II) for GO and GO/ZnO adsorbents. Metal concentrations were prepared using as reference the water quality of typical rivers contaminated in the central-northern zone of Chile. Experiments were carried out at various concentrations of Al(III) (9.0 mg/L–71.0 mg/L) (added as KAl(SO_4_)_2_·12H_2_O) and Cu(II) (3.0–24.0 mg/L) (added as CuSO_4_·5H_2_O). The experiments were performed in the first instance using the resulting pH for the solutions and later the pH was adjusted to 4 by adding 0.01 M NaOH or 0.01 M HCl. For each concentration, 20 mg of the adsorbent were added into the tubes with 40 mL of solution. Batch experiments were performed with shaking (380 rpm) at room temperature (22 °C) for 20 h. After equilibrium, aqueous phases were separated from the solids by centrifugation (6000 rpm, 5 min) and subsequently filtered through 0.22 µm membranes to finally quantify metal cations residual concentrations. The sorption capacity at equilibrium qe (mg/g sorbent) was calculated using Equation (1):

(1) $$q_{e}=\frac{\left(C_{0}-C_{e}\right)\times V}{m}$$

where $C_{0}$ is the initial concentration (mg/L), $C_{e}$ is the aqueous-phase equilibrium metal concentration (mg/L), V is the volume of suspension (L), and m is the mass of the adsorbent (g).

### 2.5. Adsorption Isotherms

The experimental data obtained in batch adsorption experiments were fitted using Langmuir and Freundlich models. The sorption capacity q (mg/g sorbent) for Langmuir, Freundlich, Tempkin and Dubinin–Radushkevich models was obtained using Equations (2)–(5), respectively [50].

(2) $$q=\frac{q_{L}K_{l}C_{e}}{1+K_{L}C_{e}},$$

(3) $$q=K_{F}C_{e}^{\frac{1}{n}},$$

(4) $$q=B\ln A_{t}C_{e},$$

(5) $$q=q_{s}\exp\left(-k_{ad}\varepsilon^{2}\right),$$

where

-$q_{L}$: amount of adsorption corresponding to a monolayer coverage;

-$K_{L}$: Langmuir constant related to the energy of adsorption;

-$K_{F}$: constant related to adsorption capacity;

-n: constant related to adsorption intensity;

-$A_{t}$: Tempkin isotherm equilibrium binding constant (L/g);

-B: short form of expression RT/b_t_, where R, T and b_t_ represent the gas constant (8.314 J/mol K), absolute temperature (K) and Tempkin isotherm constant;

-$q_{s}$: theoretical isotherm saturation capacity (mg/g);

-$k_{ad}$: Dubinin–Radushkevich isotherm constant (mol^2^/kJ^2^);

-ε: Short form of expression RT$\ln[1+\frac{1}{C_{e}}]$, where R and T represent the gas constant (8.314 J/mol) and absolute temperature (K);

-$C_{e}$: equilibrium concentration of metal in aqueous solution (mg/L).

### 2.6. Kinetic Experiments

To the kinetic experiments, 20 mg of each adsorbent was added to 40 mL of metal solution. The metal concentrations used for Al and Cu were 3674 mg/L and 2235 mg/L, respectively. The suspensions were shaken at 380 rpm at room temperature. The samples were taken at 30 min, 1 h and 22 h. The kinetic experimental data were fitted using a pseudo-first order and pseudo-second-order kinetic equations. The pseudo-first-order (6) and the pseudo-second-order (7) kinetic equations are presented as [51]:

(6) $$\log\left(q_{e}-q_{t}\right)=\log q_{e}-\frac{k_{1}}{2.303}t,$$

(7) $$\frac{t}{q_{t}}=\frac{1}{k_{2}q_{e}^{2}}+\frac{t}{q_{e}},$$

where

-$q_{e}$: adsorption capacity at equilibrium (mg/g);

-$q_{t}$: adsorption capacity at the time t (mg/g);

-$k_{1}$: constant of first-order adsorption (1/min);

-$k_{2}$:constant of second-order adsorption (g/(mg·min)).

In order to characterize the kinetic curve of the pseudo-second-order model, the approaching equilibrium factor (R_w_) was determined for each case. According to Wu et al. [52], the R_w_ equation is described as:

(8) $$R_{w}=\frac{1}{1+k_{2}q_{e}t_{ref}},$$

where $t_{ref}$: longest operation time (based on kinetic experiments)

### 2.7. Chemical Analyzes

Metal cations concentrations were measured using a UV-vis spectrophotometer (DR3900, Hach, Loveland, CO, USA) with the specific method for each element determination. Electrical conductivity (CDC401, Hach, CO, USA) and pH (PHC301, Loveland, CO, USA) were measured immediately after sample collection using a multi-meter (Hq40d Multi, Hach, Loveland, CO, USA).

### 2.8. Quality Assurance/Quality Control (QA/QC)

All analyzes and experiments were performed following QA/QC plan to obtain reliable and accurate results. All the chemicals used in this work were analytical grade. Furthermore, the accuracy and precision of the cation measurements were verified and compared with synthetic standard samples of known concentration, obtaining percentages of error less than 5%. The instruments were calibrated before being used according to the instrument guidelines. All materials were neatly cleaned and rinsed with Milli-Q water.

## 3. Results and Discussion

For this study, characterization analyzes and batch adsorption experiments were performed using GO and GO/ZnO as nanoadsorbents. Here, GO was used to evaluate the effectiveness of functionalization with ZnO nanoparticles for Al and Cu removal from water solutions. The results are presented below.

### 3.1. Adsorbent Characterization

**Figure 1:**
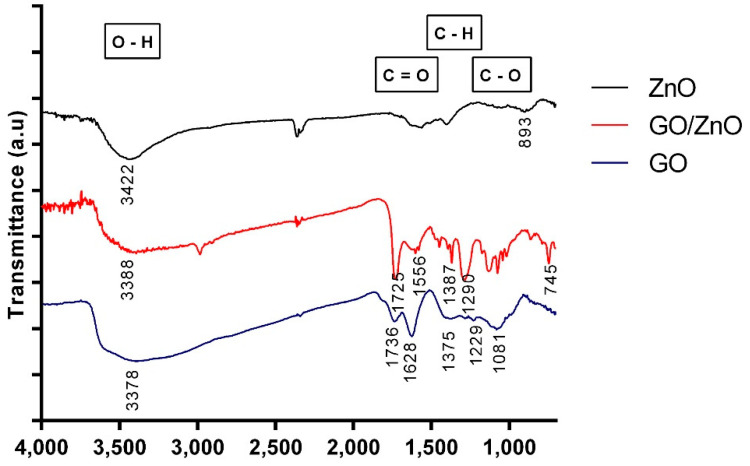
FT-IR spectrum of GO, ZnO and GO/ZnO nanoparticles.

The characterization of the nanoparticles included different techniques to study the properties of the materials before and after the functionalization process. The results were compared with data previously reported in the literature. The FT-IR spectroscopy was used to identify and characterize the chemical structure of nanoparticles studied in this work. Figure 1 shows the FT-IR spectra of GO, ZnO and GO/ZnO. For the GO sample, the absorption band at 3378 1/cm corresponds to the stretching OH-groups vibrations, which indicates the presence of hydroxyl groups in the GO layers [53]. The absorption peaks at 1736 1/cm and 1628 1/cm correspond to the C=O stretching [54]. The carbonyl signal can be related to carboxylic acids, ketones, aldehydes or esters [55]. The absorption peak at 1375 1/cm is related to C-H groups [56], while the peak at 1229 1/cm and 1081 1/cm corresponds to the C-O stretching [57]. For ZnO nanoparticles, the characteristic signals of metal oxides can be seen below 1000 1/cm, which corresponds to the fingerprint area of the IR spectrum [58]. Additionally, hydrogen tends to form hydrogen bonds with metal oxides, and it is also possible to observe the absorption band of the stretching vibration of the O-H groups at 3422 1/cm [59]. Comparing the previous spectra with the FT-IR spectrum for GO/ZnO, the characteristic band of the stretching vibration of the O-H groups can be observed at 3388 1/cm [33]. Since it decreases in thickness, it can be established that these groups are responsible for the interaction with the ZnO nanoparticles [33]. On the other hand, a series of peaks between 1000 and 1700 1/cm can be observed, which are attributable to bonds that contain oxygen. These functional groups are mainly responsible for the attractive forces between nanoparticles and metal ions [60,61]. In addition, the characteristic signals of ZnO are distinguished at 745 1/cm and 435 1/cm (data not shown) [33].

The surface area analysis using the BET method was employed to compare the surface properties of GO, ZnO and GO/ZnO nanoparticles. The ZnO nanoparticles showed higher values for BET surface area (45.58 m^2^/g), pore volume (0.38 cm^3^/g), pore diameter (30.87 nm) and sorption capacity (0.47 mmol/g) than GO (25.06 m^2^/g, 0.07 cm^3^/g, 15.06 nm and 0.26 mmol/g, respectively). These results can be explained due to the size of the ZnO nanoparticles (avg. particle size < 40 nm) and their good dispersibility in water [62], which implies a large exposed surface. Other studies also show a good dispersion of GO in water, compared to some organic solvents [63]. However, the GO decorated with ZnO nanoparticles decreases the value of its surface parameters compared to the nanoparticles separately (surface area: 4.19 m^2^/g; pore volume: 0.01 cm^3^/g; pore diameter: 7.21 nm; and sorption capacity: 0.04 mmol/g). This effect can be explained because when decorating the GO with the ZnO nanoparticles, some pores are blocked which become inaccessible, and therefore, the BET surface area decreases [64,65].

**Figure 2:**
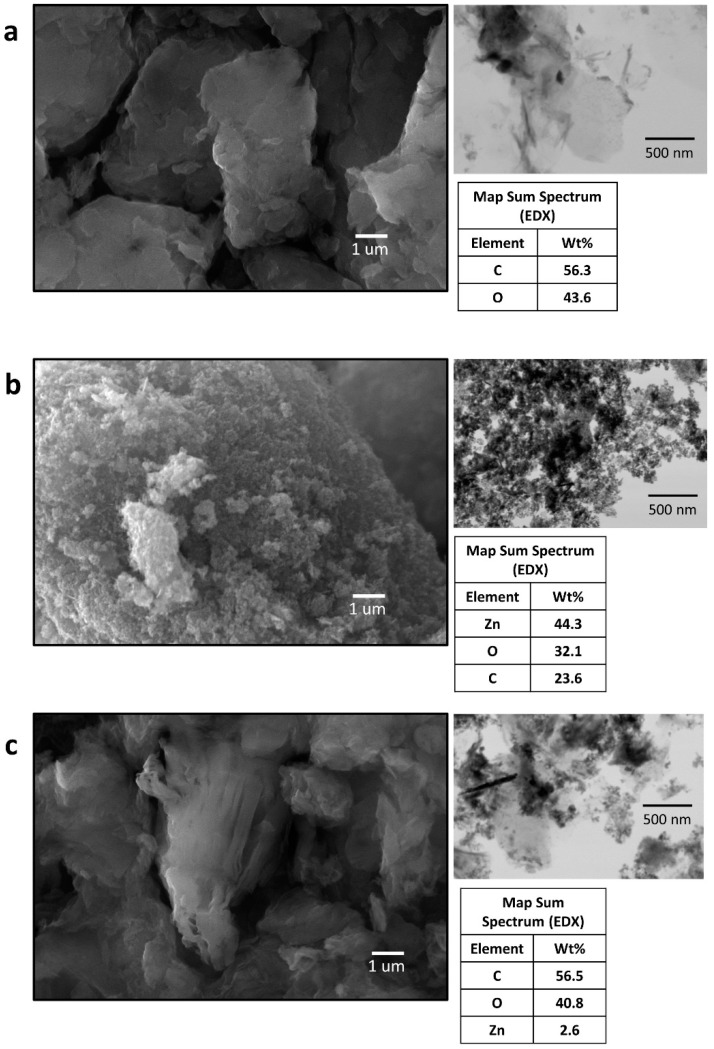
Scanning electron microscopy coupled with energy-dispersive X-ray spectroscopy (SEM-EDX) and scanning transmission electron microscopy (STEM) of (**a**) GO, (**b**) ZnO and (**c**) GO/ZnO.

The SEM micrographs of GO, ZnO and GO/ZnO are shown in Figure 2. It can be seen that the GO has a layered structure and is mainly composed of carbon and oxygen in similar proportions. Additionally, field emission scanning electron microscopy (FESEM) images showed GO nanosheets with 260 nm–730 nm range size (figure not shown). Figure 2b shows ZnO nanoparticles forming some agglomerations. These nanoparticles are mainly composed of zinc and oxygen, and the presence of carbon is also detected, which is attributable to impurities in the sample [66,67]. Finally, the STEM images of GO/ZnO (Figure 2c) show ZnO nanoparticles closely adhered to the surface of GO, and the EDX analysis shows the presence of zinc in a small fraction, corresponding to the nanoparticles added for the decoration of the GO, while the main constituent elements are carbon and oxygen. According to the GO/ZnO preparation, a 1:5 ratios by weight of ZnO and GO were used, respectively. The SEM-EDX results show an efficiency of around 16% in the decoration process, according to molecular weights. Hadadian et al. [33] reported similar compositions for zinc oxide-graphene nanocomposites, where the elemental composition percentage follows the order C > O > Zn. Furthermore, Ranjith et al. [32] presented GO/ZnO nanocomposites with 2.5% by weight of ZnO, and other authors have also shown percentages of GO functionalization lower than 2% [68].

### 3.2. Adsorption Experiments

**Figure 3:**
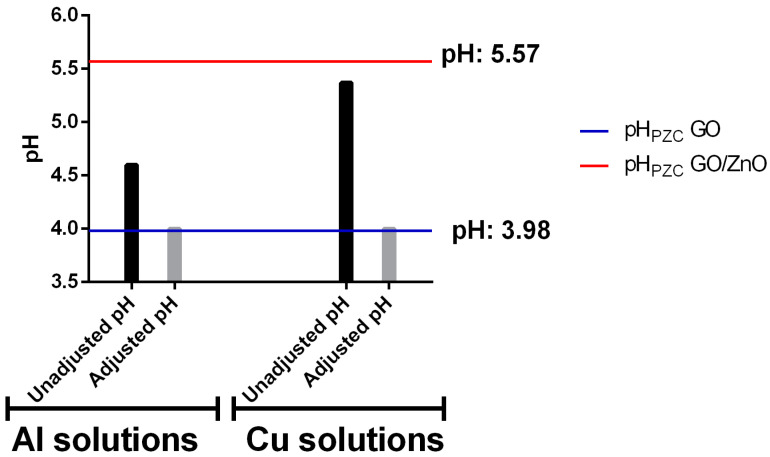
The pH values for Al and Cu monometallic solutions for experiments with and without pH adjustment. The blue line indicates the pHPZC for GO and the red line for GO/ZnO.

Adsorption experiments were carried out with and without pH adjustment to simulate different types of acidic wastewater, similarly to AMD waters. For the experiments without pH adjustment, metallic solutions were prepared with the desired concentrations of Al and Cu separately and using the resulting pH value. Instead, for the experiments with pH adjustment, the pH values were adjusted to 4, to simulate the typical conditions of sites contaminated with AMD in northern Chile. The pH values for the solutions with and without adjusting pH are shown in Figure 3. In addition, the calculated values for the pH_PZC_ are shown in horizontal lines. In this way, it is possible to observe under which conditions the pH value for the experimental solution was higher or lower than the pH_PZC_ of each adsorbent. The pH range studied in this work was also previously analyzed by Alswata et al. [35], who studied the ZnO supported on zeolite for the removal of Pb and As. They observed that the optimal pH for the adsorption process was for pH values between 4 and 5. Additionally, under these pH conditions, no ZnO leaching was evidenced. Only under more acidic conditions (pH < 4), an increase in ZnO in aqueous solution was detected, similarly to that reported by Yoshida [69], who observed leaching of ZnO at pH below 3.

The pH_PZC_ is an important parameter that indicates the pH value at which the surface is neutral. This implies that at a pH higher than this point, the surface is negatively charged, so it has an affinity for positive charges (e.g., metal ions) and vice versa [70,71]. The determination of pH_PZC_ for GO nanosheets has been carried out in other studies, obtaining results very similar to those obtained in this work, with values between 3.1 and 3.9 [72,73]. In the case of GO/ZnO nanocomposites, the material characteristics vary depending on the functionalization process. However, Zarrabi et al. [74] determined that the pH_PZC_ is 6.0 for GO/ZnO.

The monometallic Al solutions without pH adjustment were made at pH values of 4.6, while the Cu solutions presented values of 5.37. Both pH values are higher than the GO pH_PZC_ but are below than the GO/ZnO pH_PZC_. This condition should favor GO more than the GO/ZnO in the affinity for metal ions, according to the surface charge of the nanoadsorbents under these pH values. On the other hand, the experiments with pH adjustment were carried out at pH 4. This value is below the pH_PZC_ of GO/ZnO and corresponds to a pH very similar to the pH_PZC_ of GO. This pH value is lower than that obtained without pH adjustment and moves further away from the calculated pH_PZC_ for GO/ZnO, which should further reduce its metal ion removal efficiency. However, this pH should still favor GO removal as the surface should be partially negatively charged and promote cation affinity. The pH measurements of the metallic solutions after the adsorption experiments showed a slight increase of approximately 0.6 units.

Adsorption experimental data were fitted using Langmuir, Freundlich, Tempkin and Dubinin–Radushkevich isotherm models. The parameters for these models are summarized in Table 1. According to the statistical analysis of all the experimental conditions, the results presented a better fit using the Langmuir model. This model is characterized by assuming a monolayer adsorption, that is, the adsorbed layer is only one molecule in thickness, so adsorption can only occur at a finite number of sites [50]. Several studies have been carried out with GO for the removal of various pollutants, such as Ni, Cu, Zn, Co and uranium (U), among others [27,28,30]. In all these cases the Langmuir model had a better fit. Only some studies have obtained a better fit with the Freundlich model, such as the study by Xing et al. [29] for Sr removal. Moreover, the few studies that have investigated GO/ZnO nanocomposites for the removal of metal ions, such as Cu, Co, Pb, among others, have also shown a better fit for the Langmuir model [32,33,75].

**Figure 4:**
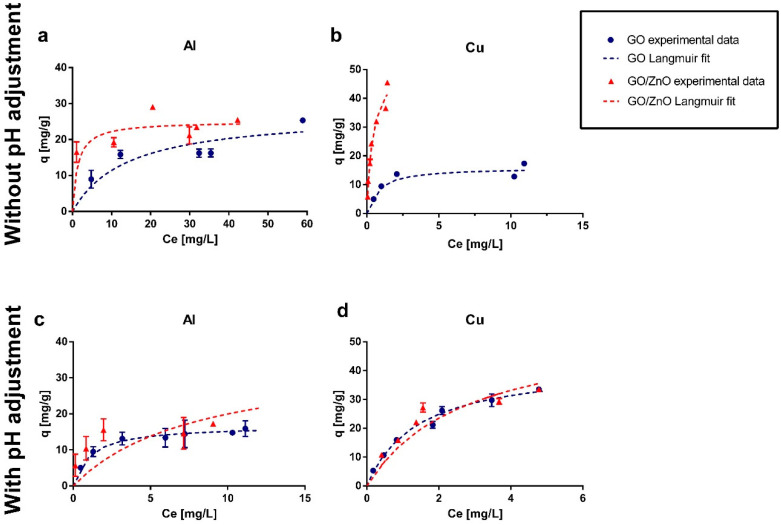
Adsorption isotherms for (**a**) Al and (**b**) Cu in experiments without pH adjustment and for (**c**) Al and (**d**) Cu in experiments with pH adjusted to 4. The standard deviation for the experimental data is also presented. In some cases, the error bars are not observed because the standard deviation is very low.

**Figure A1:**
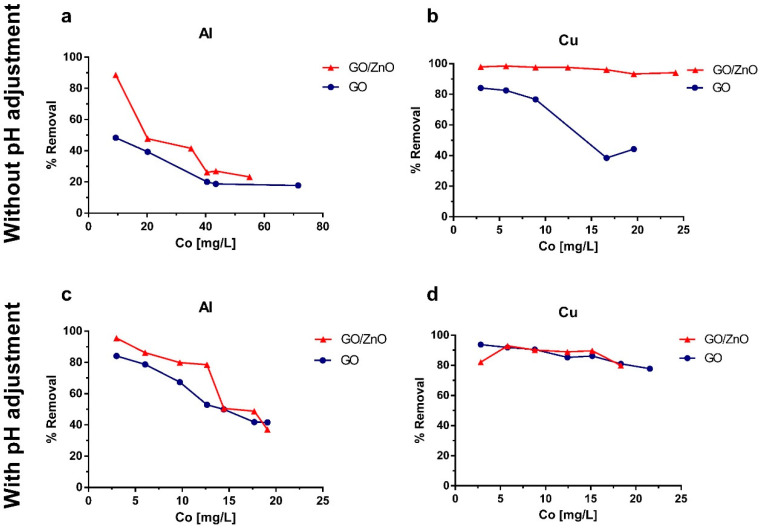
Removal percentages for (**a**) Al and (**b**) Cu in experiments without pH adjustment and for (**c**) Al and (**d**) Cu in experiments with pH adjusted to 4.

**Figure A2:**
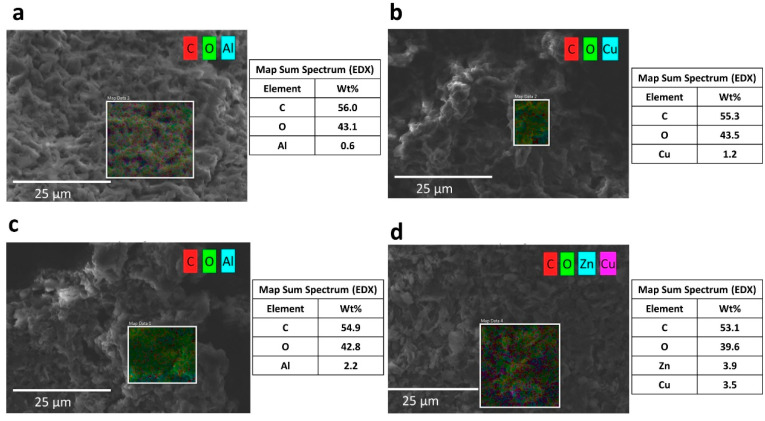
Scanning electron microscopy coupled with energy-dispersive X-ray spectroscopy (SEM-EDX) of the adsorbents after adsorption experiments: GO for (**a**) Al and (**b**) Cu removal, and GO/ZnO for (**c**) Al and (**d**) Cu removal.

Experimental results and Langmuir fit are shown in Figure 4. Additionally, in the Appendix A section, Figure A1 shows the removal percentages obtained in the experiments and Figure A2 presents the SEM-EDX analysis of the adsorbents after the adsorption experiments. From the graphs (Figure 4), it is possible to observe that without pH adjustment, the GO/ZnO reached a higher maximum sorption capacity for the removal of Al and Cu, with values of 25.48 and 45.46 mg/g, respectively, compared to the results obtained using only GO nanoparticles. In the case of pH adjusted to 4, GO/ZnO newly presented a higher maximum sorption capacity for Al removal, reaching a value of 19.9 mg/g, while GO presented a maximum value of 15.9 mg/g. Although GO/ZnO remained more efficient at this pH for Al removal, the sorption capacity decreased for both GO and GO/ZnO under more acidic conditions (Figure 4c). In contrast, Cu adsorption presented very similar removal percentages (Figure A1d) for GO and GO/ZnO, which does not allow determining conclusively which is the best Cu adsorbent under these conditions. Furthermore, control experiments were carried out using only ZnO as adsorbent material, and removal of Al and Cu greater than 90% was obtained at the different pH values studied. This accounts for the great removal efficiency of ZnO nanoparticles. However, given its size and the difficulty of completely separating it from the aqueous phase by filter media, it is necessary to impregnate it in a larger nanocomposite [33,34].

On the other hand, it should be noted that the experiments were carried out under a single temperature condition to emulate environmental treatment conditions, considering that the objective of this study focuses on the use of nanoadsorbents for environmental treatments. However, other studies have shown the effect of temperature on the sorption capacity of nanoadsorbents, particularly those based on GO. In general, it has been reported that the adsorption capacity decreases with increasing temperature because the attractive forces between the adsorbent material and the metal ion weaken at higher temperatures [28,76,77]. In general, other studies show that the best sorption capacities occur at temperatures close to 20–22 °C [76,77].

When comparing the results of Cu removal using GO with and without pH adjustment, it is possible to observe that the sorption capacity increased at a lower pH (pH 4) (Figure 4b,d). Sitko et al. [78] studied the adsorption of divalent metals using GO in a pH range of 2 to 8. They observed that the sorption capacity increased rapidly up to pH 4, reaching a removal rate of more than 90%. At higher pH values the adsorption remained constant, with a slight decrease to pH 8. On the other hand, according to Wang and Qin [79], at a pH below 5, the dominant species of copper is free Cu (II), while at a pH above 5, precipitation of insoluble metal hydroxides occurs, which restricts true adsorption studies due to simultaneous precipitation processes. This could explain why the removal rate improved at pH 4 in our experiments. However, several studies have reported that pH 5 is the optimal value for Cu adsorption [12]. Therefore, the adsorption of Cu using GO can be explained on the one hand by the surface properties, particularly the GO pH_PZC_ that produces a maximum sorption capacity at pH higher than 4 and, on the other hand, due to the speciation of the Cu as a function of pH. This effect can be observed for GO, but it was not so evident for GO/ZnO, since in both scenarios the experimental pH is below its pH_PZC_, which reduces the cation sorption capacity.

For the removal of Al, several studies have shown that the sorption capacity for different carbon-based adsorbents has an upward behavior at pH below 4 and becomes constant at pH above 4 [13,80]. However, Wang et al. [81] showed a decrease in sorption capacity by increasing the pH from 4.5 to 7.5 using reduced graphene oxide. On the other hand, Rajamohan et al. [82] found a plateau between pH 4.5 and 6, values at which the sorption capacity is maximum. From these data it is possible to infer that the range of pH studied in this work is within the pH values where Al adsorption is optimal, Thus, the differences between removal efficiencies could be attributable to the type of adsorbent used and its surface characteristics, more specifically, to the pH_PZC_ values.

Finally, it can be observed that GO/ZnO presented better removal percentages for Al at lower concentrations, while when increasing the initial concentration, a saturation in the material is observed, since the sorption capacity (mg/g) became more constant. On the other hand, for Cu, a high removal rate is observed at the different initial concentrations studied. This can also be explained due to the speciation of Al and Cu as a function of pH. While Cu remains as a stable divalent ion at a pH lower than 5 [12,83], Al undergoes more changes in speciation in the studied pH range, being able to find different partial charges (Al(OH)_3_, Al(OH)_2_^+^, Al(OH)^2+^, Al^3+^) [84], which could explain that the removal of this metal was lower than in the case of Cu. Furthermore, Liu et al. [85] studied the adsorption of the divalent (Pb^2+^, Cd^2+^, Cu^2+^) and the trivalent (Cr^3+^) ions onto titanate nanotubes, and they found that the trivalent ion was less adsorbed than the divalent ions studied, possibly due to the effects of hydrated ion radius and hydration energy of ionic metals. This implies that the efficiency of metal removal is not controlled solely by the properties of the material, such as pH_PZC_, but there are other factors specific to each pollutant that determine the ability to be adsorbed on a certain surface.

### 3.3. Kinetic Experiments

From the experimental data in kinetic experiments, it was possible to determine that during the first 30 min the removal percentage is greater than 50% of the total removal percentage, while after one hour the removal is greater than 70% of the total removal (data not shown). This provides evidence of rapid removal kinetics. Previous studies of graphene as an adsorbent for pollutants, such as U, Zn, Co and Cd, have investigated sorption kinetics. In general, studies show that equilibrium is reached at approximately 30 min for the different types of adsorbates [30,76,86]. White et al. [28] studied Cu adsorption using GO nanoparticles obtaining equilibrium times close to 40 min. In the case of ZnO, some kinetic studies have been carried out for the removal of some pollutants such as Pb, Cd or Co, obtaining equilibrium times of 100 min or less [87,88].

**Figure 5:**
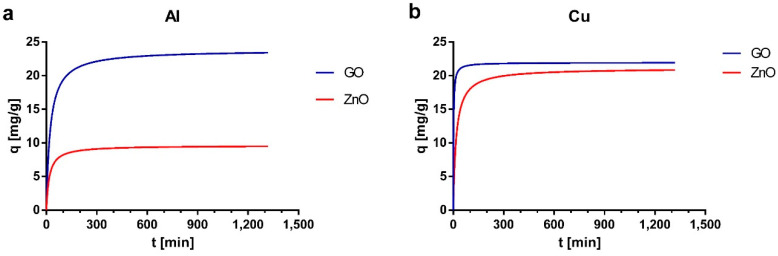
Kinetic curves for (**a**) Al and (**b**) Cu based on the pseudo-second-order model.

Experimental data were fitted with pseudo-first-order and pseudo-second-order kinetic models. The kinetic adsorption parameters for both models are shown in Table 2. According to the values of the coefficient of determination (R^2^) and the theoretical adsorption capacities associated with each model (q_e1_ and q_e2_), it was determined that the model that presents a better fit is the pseudo-second-order. Additionally, the kinetic curves are presented in Figure 5, in which the sorption capacity of the materials can be observed as a function of time and the rapid saturation time that they present. The pseudo-second order kinetic model is the most used to fit velocity data for the adsorption of metal ions and other pollutants [89]. The number of publications using this model is almost three times higher than those using other models such pseudo-first-order or intraparticle diffusion models [52]. Studies have shown that the adsorption behavior that is well described by the pseudo-second order model is mainly explained by diffusion-based mechanisms [89].

The approaching equilibrium factor (*R_w_*) was determined for each kinetic experiment. The *Rw* factor describes the type of kinetic curve and indicates the proximity to the equilibrium level of the system, which accounts for the effectiveness of the adsorption curve in the time studied [52]. Table 3 summarizes this information. In general, the curves obtained are very close to equilibrium, which ensures good performance in the adsorption experiments of the next section with the selected contact times.

## 4. Conclusions

In this study, our preliminary results showed that the GO decorated with ZnO nanoparticles improves the performance of GO as an ionic metal adsorbent for its potential use in AMD remediation. The SEM-EDX results show the presence of ZnO nanoparticles on the GO surface after the functionalization process. The pH_PZC_ of GO/ZnO is considerably higher than that of non-functionalized GO, which means a disadvantage for GO/ZnO in cation adsorption processes at low pH due to positive surface charge. The Al and Cu removal was studied in two experimental conditions: with and without pH adjustment. In both cases, the pH was below the pH_PZC_ of GO/ZnO. However, the removal capacity of both metals was equal or higher for GO/ZnO than for GO, even under acidic conditions, which shows the effectiveness of functionalization with ZnO nanoparticles to improve the adsorption capacity for Cu and to a lesser extent for Al. Even though the improvement in adsorption capacity after decoration with ZnO is not radical, a slight improvement was observed with the incorporation of ZnO nanoparticles, a nanoadsorbent that despite showing a great adsorption capacity on its own, is limited to its use in acidic waters due to its size and its high pH_PZC_ value. This value presents an intermediate value between the two adsorbents after the functionalization process. Thus, the studied pH conditions revealed the differences between the performance of the adsorbents at pH values close to GO pH_PZC_, a critical parameter in the adsorption effectiveness of this material.

Our preliminary findings contribute to a deeper understanding of the potential and effectiveness of GO functionalization with ZnO nanoparticles for the removal of heavy metals, particularly Al and Cu, typical AMD pollutants. The synthetic waters prepared in this study constitute a simplified approach that emulates different pH conditions to obtain a preliminary approach to the possible application of this nanoadsorbent for the treatment of real waters contaminated with AMD. Additional efforts should be made in the study of adsorbent desorption processes for the recovery of materials and to achieve an approach to the use of these materials in real treatment systems. Furthermore, the effect of competition for adsorbent material binding sites between the different ions present in real AMD waters should be studied.

## Figures and Tables

**Table 1:** Parameters for the Langmuir, Freundlich, Tempkin and Dubinin-Radushkevich isotherm models for experiments with and without pH adjustment.

Experimental Condition	Sample	Metal	Langmuir			Freundlich			Tempkin			Dubinin–Radushkevich		
			*q_L_* (mg/g)	*K_L_* (L/mg)	R^2^	*K_F_* (L/g)	*n*	R^2^	*A_T_* (L/g)	*B*	R^2^	*q_s_* (mg/g)	*k_ad_* (mol^2^/kJ^2^)	R^2^
Without pH Adjustment	GO	Al	27.40	0.07	0.858	2.12	2.97	0.835	1.23	5.10	0.766	21.57	−0.0019	0.808
		Cu	15.97	1.33	0.947	2.49	3.39	0.737	21.32	2.91	0.762	17.59	−0.0004	0.898
	GO/ZnO	Al	25.06	0.78	0.963	3.36	8.73	0.598	982.39	2.35	0.511	24.14	−0.0002	0.547
		Cu	53.48	2.40	0.962	4.86	1.77	0.937	27.17	11.24	0.968	28.94	−0.00005	0.496
With pH Adjustment	GO	Al	16.78	0.91	0.995	2.41	3.06	0.909	13.29	3.16	0.961	16.71	−0.0004	0.990
		Cu	42.37	0.71	0.985	3.29	1.81	0.979	8.49	8.67	0.974	37.87	−0.0004	0.988
	GO/ZnO	Al	36.10	0.12	0.980	3.23	5.42	0.681	228.91	2.21	0.570	17.59	−0.0002	0.856
		Cu	44.05	0.79	0.908	2.84	1.28	0.233	4.97	11.04	0.853	49.11	−0.0006	0.731

**Table 2:** Kinetic adsorption parameters for pseudo-first-order and pseudo-second-order models.

Sample	Metal	Initial Concentration (mg/L)	$q_{e}^{exp}$ (mg/g)	Pseudo-First-Order			Pseudo-Second-Order		
				*k*_1_ (1/min)	*q*_*e*1_ (mg/g)	*R* ^2^	*k*_2_ (g/mg min)	*q*_*e*2_ (mg/g)	*R* ^2^
GO	Al	36.74	23.4	0.0088	2.91	0.9998	0.0018	23.81	1.0000
	Cu	22.35	21.92	0.0140	0.99	0.9885	0.0262	21.93	1.0000
ZnO	Al	36.74	9.5	0.0115	1.74	0.9874	0.0061	9.62	0.9965
	Cu	22.35	20.82	0.0120	2.32	0.9933	0.0028	21.10	0.9998

**Table 3:** The approaching equilibrium factor (Rw) in the pseudo-second-order kinetic model.

Sample	Metal	*q_e_* (mg/g)	*k*_2_ (g/mg min)	*t_ref_* (min)	*R_w_*	Type of Kinetic Curve	Approaching Equilibrium Level
GO	Al	23.81	0.0018	1320	0.0174	Largely curved	Well approaching equilibrium
	Cu	21.93	0.0262	1320	0.0013	Pseudo-rectangular	Drastically approaching equilibrium
ZnO	Al	9.62	0.0061	1320	0.0127	Largely curved	Well approaching equilibrium
	Cu	21.10	0.0028	1320	0.0127	Largely curved	Well approaching equilibrium
